# Nutritional enrichment of broiler breast meat through dietary supplementation of Indian ginseng *Withania somnifera* and synbiotic substances under semi-arid climatic conditions

**DOI:** 10.14202/vetworld.2017.1301-1306

**Published:** 2017-11-03

**Authors:** Sonal Thakur, Tribhuwan Sharma, Radhe Shyam Arya, Basant Bais, Vijay Kumar Agrawal

**Affiliations:** 1 Department of Animal Nutrition, College of Veterinary and Animal Sciences, Rajasthan University of Veterinary and Animal Science, Bikaner - 334 001, Rajasthan, India; 2 Department of Livestock Production and Technology, College of Veterinary and Animal Sciences, Rajasthan University of Veterinary and Animal Science, Bikaner - 334 001, Rajasthan, India; 3 Department of Animal Genetics and Breeding, College of Veterinary and Animal Sciences, Rajasthan University of Veterinary and Animal Science, Bikaner - 334 001, Rajasthan, India

**Keywords:** breast meat, broiler, heat stress, synbiotics, *Withania somnifera*

## Abstract

**Aim:**

The present study was conducted to explore the effect of supplementation of *Withania somnifera* and synbiotics alone or in combination on the composition of broiler breast meat under heat stress conditions.

**Materials and Methods:**

A 42-day feeding trial was conducted on 360 broiler chicks randomly allotted into eight treatment groups with three replicates each under completely randomized design. The T_1_ group was kept as control whereas T_2_-T_4_ were supplemented with 0.5%, 1%, and 1.5% *W*. *somnifera* root powder; T_5_ and T_6_ were supplemented with 0.025% and 0.050% synbiotic and T_7_ and T_8_ were fed on diet containing 0.25% *W*. *somnifera*+0.025% synbiotic and 0.50% *W*. *somnifera*+0.05% synbiotic, respectively. Three broilers from each replicate were sacrificed at the end of the trial to estimate crude protein (CP), ether extract and ash content of the breast muscle on dry matter basis.

**Results:**

Significantly higher CP values and lower ether extract values were observed in 1.5% *W*. *somnifera* supplemented group (T_4_) or in group supplemented with 0.50% *W*. *somnifera* and 0.05% synbiotic (T_8_). The ash content of breast meat was observed non-significant in T_1_-T_4_ groups however the inclusion of synbiotics in T_5_-T_8_ groups significantly raised the ash contents.

**Conclusion:**

The study concluded that inclusion of 0.5% *W. somnifera* with 0.05% synbiotic substance enriches the total protein content and reduces the total lipids content of broiler breast meat under heat stress conditions.

## Introduction

Poultry meat is an excellent source of high-quality protein, vitamins and minerals for human nutrition. India ranks 5^th^ in broiler meat production and contributes nearly 2.53% of world’s chicken meat production [1]. The poultry sector contributes 1% of gross domestic product (GDP) and 11% of livestock GDP in India. Huge economic losses often occur in broiler industries through decreased consumer acceptance, transportation and storage losses caused by deterioration in meat quality under higher ambient temperature and erratic climatic pattern. The impact of global warming has further worsened the situation. The semiarid region of Bikaner (Rajasthan) is known for its harsh, extreme climate with scanty and erratic rainfall.

The breast muscle is well developed by genetic selection for rapid growth and carcass yield and constitute about 22-25% of the whole carcass weight [2]. The production of quality breast meat is affected by the environmental stress factors. Heat stress induces oxidative damage of tissues and this result with alteration of the chemical composition and also sensory quality of chicken meat [3]. Heat-stressed broilers produce pale, soft, and exudative breast meat with lower protein and ash content [4]. The crude protein (CP) content of breast meat decreases markedly (p<0.05) with a significant increment in crude fat as the ambient temperature exceeds 33°C with or without high relative humidity [5]. Baziz *et al*. [6] reported that the heat-stressed broilers had a higher level of abdominal fat compared to broilers raised under normal temperature. Broiler such as Vencobb strain developed for faster growth and production is particularly more vulnerable to environmental stress due to their greater metabolic activity and more body heat [7].

Reduced feed intake, impaired growth performance, higher feed conversion ratio, reduced dietary digestibility, decreased plasma protein, and calcium levels have been reported in broilers that were subjected to chronic heat stress [8]. The quality of broiler meat is affected by temperature-associated environmental challenges as broilers are devoid of sweat glands and are fully covered with feathers [9]. The carcass quality of broiler meat reduces rapidly when the temperature humidity index (THI) exceeds the thermal comfort zone [10]. The production of quality poultry meat without any chemical residues in an economic manner is also an order of the day.

Several methods have been suggested to maintain the quality of broiler meat through alleviation of negative effects of high environmental temperature [11]. The *W. somnifera* or Indian Ginseng root powder is one such well known anabolic, hypolipidemic, antistressor, and antioxidant phytoherb [12] having homeostatic role in the body. The alkaloids found in the roots of *W. somnifera* such as withaferin and withanolides are responsible for most of its biological properties [13]. Limited information suggests that an organic growth promoter substance like synbiotics could provide additive benefits in growth performance [14] through increase digestibility and availability of proteins, vitamins, and mineral elements [15].

Thus, the present study was undertaken to observe the anabolic and hypolipidemic effect of *W. somnifera* on broiler breast meat composition and the nutrient sparing effect of synbiotics in heat-stressed broilers during the transition month of March-April under semi-arid climatic condition.

## Materials and Methods

### Ethical approval

An approval from the Institutional Ethical Committee was obtained before the onset of experimental trial.

### Procurement of experimental material

360-day-old unsexed Vencobb broiler chicks of similar body weight and in good health condition were procured from commercial hatchery (M/s Kewalramani Hatcheries, Ajmer, Rajasthan, India). The *W*. *somnifera* root powder (M/s Mohanlal and Sons Pvt., Ltd., Bikaner, Rajasthan, India) and synbiotic substances (Polchem Pharma, Pune, Maharashtra, India) in sufficient quantity were procured. The readymade broiler starter and finisher feed in mash form were procured from reputed poultry feed manufacturer (Venkys Pvt., Ltd., Ajmer, Rajasthan, India). The proximate composition of broiler starter, broiler finisher, *W*. *somnifera*, and synbiotic is presented in Table-1. The ingredient composition of synbiotic substance is presented in Table-2.

**Table-1:** Proximate analysis^1^ of broiler feed, *Withania somnifera* and synbiotic substances.

Chemical composition	Broiler starter	Broiler finisher	*Withania somnifera*	Synbiotic mixture
Proximate principles (%) (dry matter basis)				
Moisture	7.0	7.0	4.88	3.96
Organic matter	93.0	93.0	95.12	96.04
Crude protein	22.87	20.1	5.97	23.43
Ether extract	5.8	6.9	0.65	0.5
Crude fiber	3.95	4.36	13.8	3.5
ME (kcal/kg feed)	2900	3100	-	-
Mineral composition (%)				
Calcium	1.02	1.06	1.17	0.83
Phosphorus	0.96	0.81	0.63	1.28

1 Average of the values determined on samples compounded on three occasions

**Table-2:** Active principles present in synbiotic used in the experimental trial.

Ingredients	Active constituents	Concentration
Prebiotic	Mannan-oligosaccharide	14-16%
Probiotics	*Lactobacillus acidophilus*	10^9^ CFU/g
	*Lactobacillus bulgaricus*	
	*Lactobacillus plantarum*	
	*Streptococcus faecium*	
	*Bifidobacterium bifidum*	

### Experimental design

Completely randomized design was adopted in the present study. The broilers were randomly divided into eight treatment groups (T_1_-T_8_) with three replicates of 15 chicks each. The T_1_ group was kept as control without any supplementation whereas T_2_, T_3_, and T_4_ were supplemented with 0.5%, 1%, and 1.5% *W*. *somnifera* root powder; T_5_ and T_6_ were supplemented with 0.025% and 0.050% synbiotic and T_7_ and T_8_ were fed on diet containing 0.25% *W*. *somnifera*+0.025% synbiotic and 0.50% *W*. *somnifera*+0.05% synbiotic, respectively.

### Feeding and management

A 42-day experimental feeding trial was conducted in the experimental unit section of Poultry Farm of College of Veterinary and Animal Sciences, Bikaner, Rajasthan, under standard feeding and managemental conditions with broiler starter (0-21 days) and broiler finisher (21-42 days) ration. The chicks were vaccinated against New Castle Disease and Infectious Bursal Disease at the age of 4^th^ and 14^th^ day, respectively. All the birds were maintained in deep litter pens under natural cross ventilated conditions, and standard managemental practices were adopted throughout the trial to reduce systemic errors. The stocking space of 550 cm^2^ and 800 cm^2^ per broiler was provided at 0-4 weeks and 4-6 weeks of age, respectively. 23-h lighting schedule was maintained with free access to feed and water during the whole trial.

### Collection of weather information

The weekly weather information related to ambient temperature and relative humidity was collected from the Meteorological Department, Agricultural Research Station of Swami Keshwanand Rajasthan, Agricultural University, Bikaner, Rajasthan (Table-3).

**Table-3:** Ambient temperature, relative humidity and THI observed during different weeks of experimental trial.

Weeks of trial	Ambient temperature (ºC)			Relative humidity (%)			THI		
		
	Min	Max	Avg	Min	Max	Avg	Min	Max	Avg
Week-1	13.00	36.60	33.00	45.00	96.00	83.43	56.28	91.81	88.18
Week-2	12.50	34.60	29.83	47.00	87.00	80.28	55.28	90.42	82.73
Week-3	13.40	36.60	34.59	22.00	83.00	76.14	62.31	91.94	89.55
Week-4	11.00	39.40	35.12	13.00	82.00	63.86	64.07	90.41	87.60
Week-5	16.40	42.40	38.40	14.00	58.00	43.28	60.31	93.34	87.96
Week-6	18.20	37.40	36.57	23.00	67.00	54.71	63.19	90.18	88.11

THI: Temperature humidity index

### Parameters studied

Three broilers from each replicate were sacrificed at the end of trial with minimum stress and discomfort as per standard procedure [16]. The breast meat portion was harvested after evisceration and kept under refrigerated condition at 4°C for 24 h. Breast meat samples were analyzed for total CP, ether extracts and ash content as per standard Association of Official Analytical Chemists Procedure [17] and were expressed on dry weight basis.

### Statistical analysis

The THI values for different weeks were calculated as per formula suggested by Kelly and Bond [18].

THI = Ta − (0.55−0.55XRH) × (Ta−58.8)

Where Ta=ambient temperature in Fahrenheit and RH=Relative humidity divided by 100.

One-way analyses of variance were performed using the general linear model procedure of SPSS software version 20.0 to test the effect of different level of *W*. *somnifera* and synbiotic alone or their combination (T_1_-T_8_) on breast meat quality. Means of different groups were compared using Duncan’s multiple range tests.

## Results

**Figure-1:**
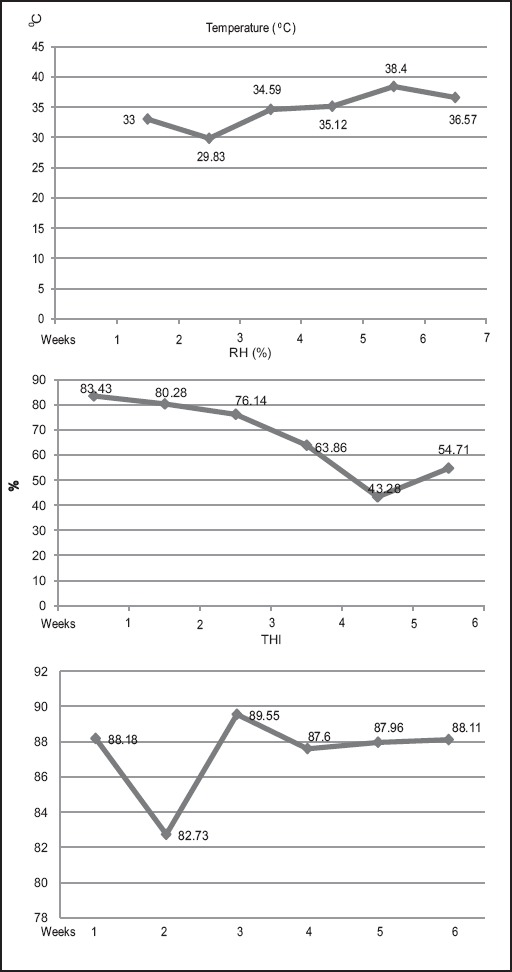
Temperature, relative humidity, and temperature humidity index trend over the weeks.

The assessment of heat stress condition on broiler during the trial was carried out and the values and trends of mean weekly temperature, relative humidity and THI are depicted in Figure-1. The mean weekly temperature was observed to be higher in all the weeks except 2^nd^ week with erratic nature of temperature fluctuation was observed. The broilers were exposed to gradually declining relative humidity till 4^th^ week that suddenly dipped in the 5^th^ week and again increased in the last week of study. The erratic pattern of climate change in terms of temperature and humidity particularly during the 5^th^ and 6^th^ weeks suggests the effect of heat stress on the body condition of broilers. However, continuous high THI value above thermal comfort zone except 2^nd^ week of trial indicates the high level of environmental stress during the whole trial period.

**Figure-2:**
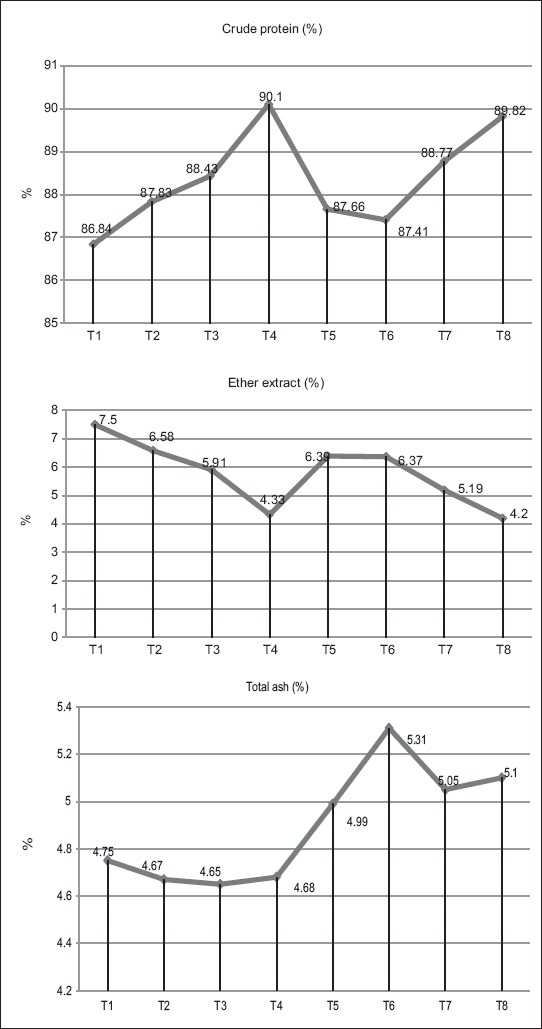
Trends in crude protein, ether extract and total ash content in broiler breast meat over the treatments. T_1_: Control, T_2_: 0.5% *Withania somnifera*, T_3_: 1.0% *W. somnifera*, T_4_: 1.5% *W. somnifera*, T_5_: 0.025% synbiotic, T_6_: 0.050% synbiotic, T_7_: 0.25% *W.somnifera*+0.025% synbiotic, and T_8_: 0.50% *W. somnifera* +0.05% symbiotic.

The proximate composition of broiler breast meat (Table-4) revealed significant (p<0.05) difference in mean CP content overall the treatments. The mean CP (dry matter basis) was highest in the broiler group supplemented with 1.5% *W*. *somnifera* however comparable performance was observed in T_8_ group supplemented with 0.5% *W*. *somnifera*+0.05% synbiotic. The CP content in different treatments ranged from 86.84% to 90.10% with lowest value observed in non-supplemented broiler group (T_1_). Mean ether extract of breast meat also exhibited significant (p<0.05) differences among the treatment groups. A downward trend in ether extract value was observed with an increased level of *W*. *somnifera* supplementation (Figure-2). The combined feeding approach through *W. somnifera* and synbiotic supplementation at 0.5% and 0.05% level, respectively, showed the lowest ether extract value. The results indicate the health promoting synergistic effect of herb *W*. *somnifera* and synbiotic substances in reduction of fatty substances. The highly significant ether extract value was observed in the control group (T_1_).

**Table-4:** Breast meat composition of broiler chicken.

Parameters	Treatment groups								SEM	p value

	T_1_	T_2_	T_3_	T_4_	T_5_	T_6_	T_7_	T_8_		

	C	0.5% WS	1.0% WS	1.5% WS	0.025% Syn	0.05 % Syn	0.25% WS+0.025% Syn	0.5% WS+0.05% Syn		
Crude protein	86.84^a^	87.83^bc^	88.43^cd^	90.10^e^	87.66^abc^	87.41^ab^	88.77^d^	89.82^e^	0.297	0.035
Ether extract	7.50^d^	6.58^c^	5.91^c^	4.33^a^	6.39^c^	6.37^c^	5.19^b^	4.20^a^	0.236	0.032
Ash	4.75^a^	4.67^a^	4.65^a^	4.68^a^	4.99^ab^	5.31^b^	5.05^ab^	5.10^ab^	0.141	0.047

C=Control, WS=*Withania somnifera,* Syn=Synbiotic, SEM=Standard error of mean

The ash content of the breast muscle was found to be highest in 0.05% synbiotic supplemented group (T_6_) whereas the treatment groups T_5_, T_7_, and T_8_ showed comparable ash content. Comparable ash content was observed in control and *W*. *somnifera* supplemented groups (T_1_-T_4_).

## Discussion

The findings pertaining to CP and ether extract content of the broiler breast meat revealed the gradual enrichment of protein and decline in the lipid content of the breast meat on the supplementation of *W. somnifera* root powder to broilers under heat stress conditions supports the anabolic effect of *W. somnifera* root powder as claimed in traditional Indian medicine [19]. The inclusion of synbiotic with *W. somnifera* root powder in a basal diet of broilers in treatment group T_8_ improved the protein content of the breast muscle which could be due to enhanced absorption of amino acids in the gut [20]. The broilers in the non-supplemented group (T_1_) showed lowest CP content in breast meat through decreased protein synthesis [3] under state of high ambient temperature. Depression in chemical composition of meat in terms of low protein and higher fat observed in breast meat of broilers in control group (T_1_) is in line with the findings of Akit *et al*. [21] who also observed 8% reduction in breast meat protein when the broilers were exposed to 34°C after 3 weeks of age. The higher ash values observed in the synbiotic supplemented groups might have occurred due to contributory effect of synbiotic. The difference in the ultimate nutritive quality of broiler meat depends on the supplementation of herbs and the severity of the thermal stress [22]. Similar investigation on *Berberis lycium* herb proved the hypolipidemic effect of herbal supplementation in boilers [23]. Likewise, Vencobb broilers raised on 1% *W*. *somnifera* root powder in basal diet exhibited anabolic effect in terms of improved body weight [24].

## Conclusion

The present study concluded that inclusion of *W. somnifera* root powder in the ration of broilers significantly increased the CP content and lowered the ether extract content of the breast meat. The broilers under the *W. somnifera* supplemented groups (T_2_-T_4_, T_7_, and T_8_) significantly showed the enhanced performance in terms of CP and fat content in breast meat than control group. The results indicate the production of protein rich low fat containing broiler breast meat suitable for the health of human consumers. Thus, the anabolic and hypolipidemic effect of *W. somnifera* root powder could be used for the production of designer broiler meat with low fat and high protein that may fetch more prices to producers.

## Authors’ Contributions

ST carried out the study. TS planned, designed and supervised the experiment, RA and BB provided technical support, VKA carried out statistical analysis. All authors read and approved the manuscript.
